# Endovascular Aortic Repair in Traumatic Descending Thoracic Aortic Transection: A Case Report

**DOI:** 10.7759/cureus.68787

**Published:** 2024-09-06

**Authors:** Nikita Changlani, Binay K Panjiyar, Saroj K Jha, Sanam W Khan, Akshita Kaushal, Sreeja Cherukuru, Diksha Mahendru, Safa Kaleem

**Affiliations:** 1 Internal Medicine, Paul L. Foster School of Medicine, El Paso, USA; 2 Cardiology, Harvard Medical School, Boston, USA; 3 Internal Medicine, California Institute of Behavioral Neurosciences & Psychology, Fairfield, USA; 4 Research, Harvard Medical School, Boston, USA; 5 Research Fellowship, Ventolini’s Lab, Texas Tech University Health Sciences Center, Odessa, USA; 6 Internal Medicine, Tribhuvan University Teaching Hospital, Kathmandu, NPL; 7 Internal Medicine, Combined Military Hospital Lahore Medical College, Lahore, PAK; 8 Internal Medicine, Dr. Rajendra Prasad Government Medical College, Kangra, IND; 9 Psychiatry, Sri Venkateshwaraa Medical College, Tirupati, IND; 10 Internal Medicine, Crozer-Chester Medical Center, Upland, USA; 11 Medicine, Shadan Institute of Medical Sciences, Hyderabad, IND

**Keywords:** aortic repair, aortic transection, case report, endovascular aortic repair, interventional cardiology

## Abstract

Aortic transection, a near-complete tear through the layers of the aorta, is a critical condition often resulting from trauma such as motor vehicle collisions. The urgency of managing aortic transection underscores the critical need for effective interventions. We report the case of a male in his early 50s with no significant medical history who presented to the emergency department following a motor vehicle collision, sustaining multiple injuries including a descending thoracic aortic transection. Rapid diagnostic assessment confirmed the severity of the injury, necessitating immediate intervention. Endovascular aortic repair was successfully employed, highlighting its efficacy in managing acute aortic injuries. The patient responded well to treatment, underscoring the importance of timely intervention in improving patient outcomes. This case emphasizes the critical role of rapid diagnostic assessment and endovascular intervention in managing life-threatening thoracic aortic injuries, particularly in the acute setting.

## Introduction

Blunt traumatic aortic injury is the second most common cause of death due to trauma after head injury [1]. The hallmark of a traumatic aortic injury is damage to the artery wall or a rupture resulting from blunt force or penetrating trauma [2]. The mechanism of damage in most cases is high-intensity blunt trauma with fast deceleration commonly linked to multiple rib fractures. Damage can occur anywhere along the aorta, generally from the ascending aorta to the iliac bifurcation, or along the descending aorta, close to the aortic isthmus [3]. Patients who survive this potentially fatal injury typically have partial thickness aortic wall tears along with the development of pseudoaneurysms [4]. Therefore, prompt diagnosis and immediate intervention are required. Several imaging modalities can be considered such as chest X-ray, chest CT, and focused assessment with sonography in trauma (FAST) scan [2]. The use of endovascular aortic repair (EVAR) has become increasingly prevalent due to its minimally invasive nature and effectiveness [5]. This case report highlights a case of traumatic descending thoracic aortic transection in a 50-year-old man managed successfully with EVAR, discusses the clinical decision-making process in the management of such injuries, and demonstrates its potential as a life-saving intervention.

## Case presentation

A 50-year-old male with no prior medical history presented to the emergency department with a trauma code after a motor vehicle collision. He was a restrained front passenger in a vehicle traveling approximately 70 mph when it was struck on the passenger side by a semi-truck. Initially ambulatory on the scene, the patient subsequently developed tachycardia and hypotension en route to the hospital. He sustained multiple injuries, including a descending thoracic aortic transection with a 1.5 cm pseudoaneurysm and periaortic hematoma 1 cm distal to the left subclavian artery, right medial rib fractures 6-10 nondisplaced, right lateral rib fractures 4-10 nondisplaced, right acetabular fracture, right pubic symphysis fracture, right inferior pubic rami fracture, and a 10 cm × 5 cm pelvic sidewall hematoma.

**Figure 1:**
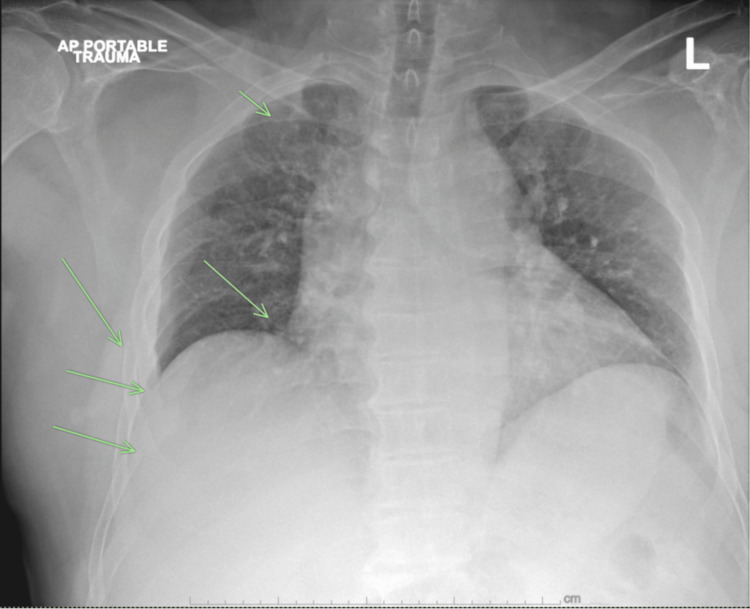
Chest X-ray demonstrating multiple right rib fractures (green arrows) and mediastinal widening.

**Figure 2:**
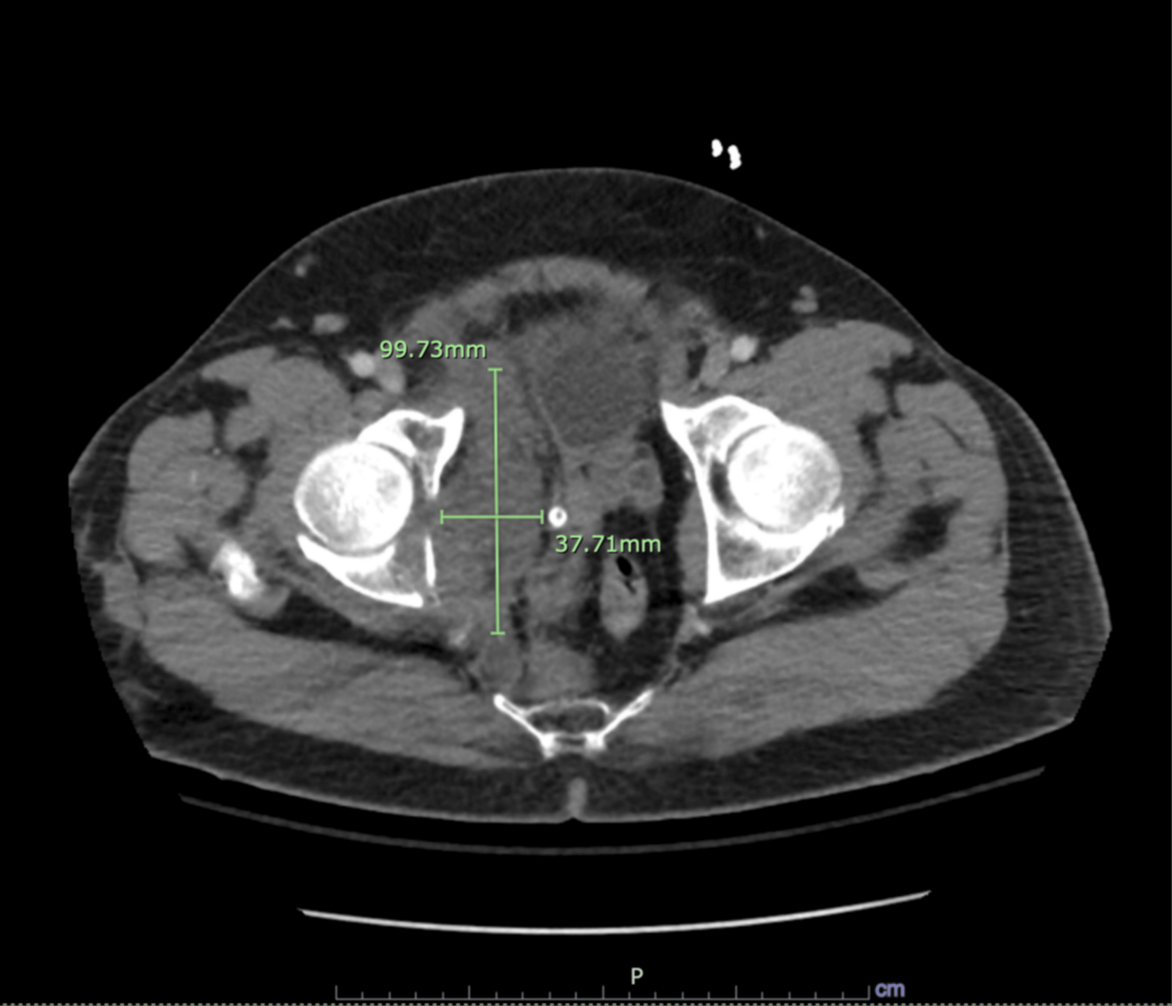
CT scan showing acute thoracic aortic injury, pseudoaneurysm, and associated periaortic hematoma.

**Figure 3:**
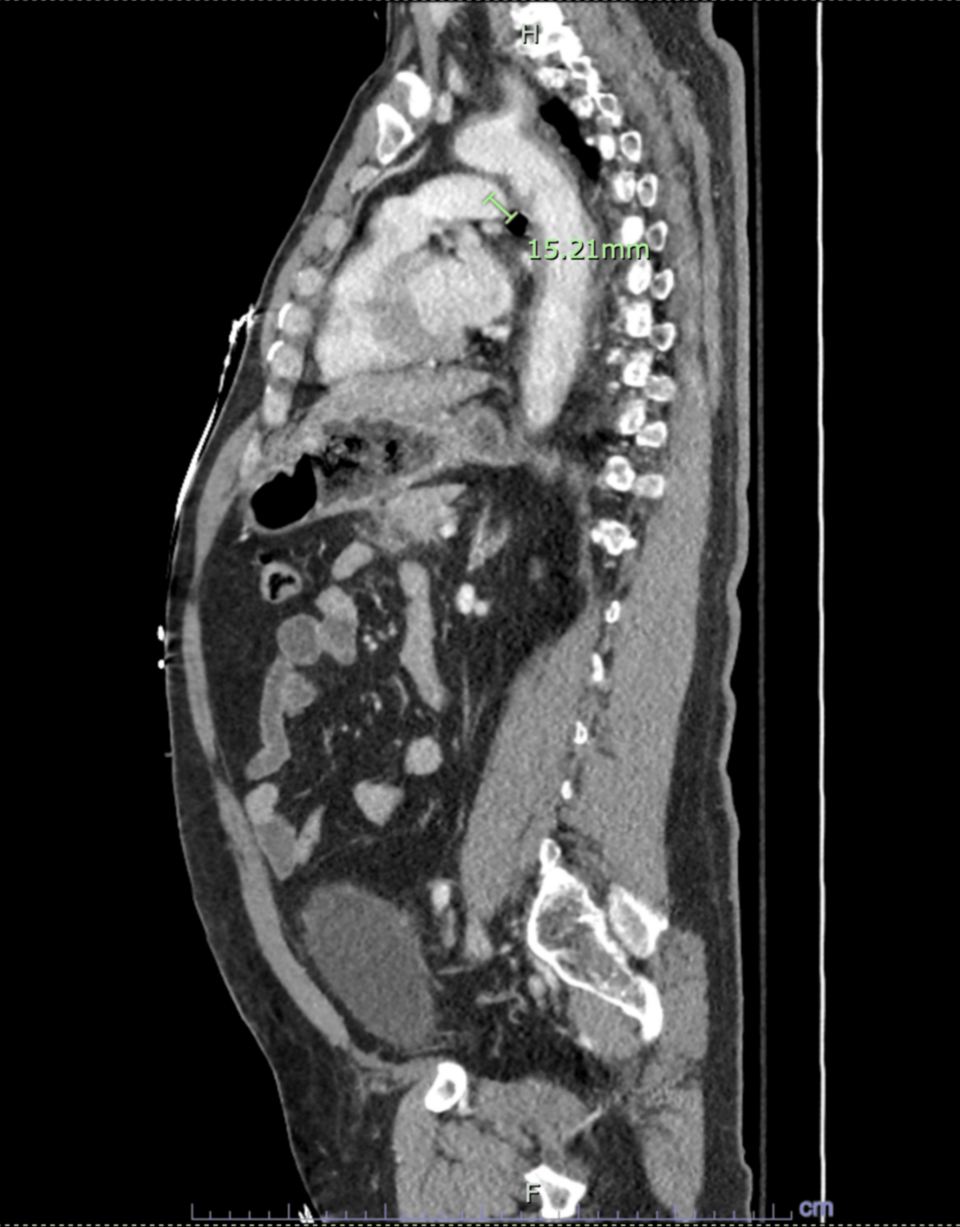
CT scan revealing a flap coursing across the medial wall of the descending aorta with an associated pseudoaneurysm measuring 1.5 cm protruding anteromedially.

**Figure 4:**
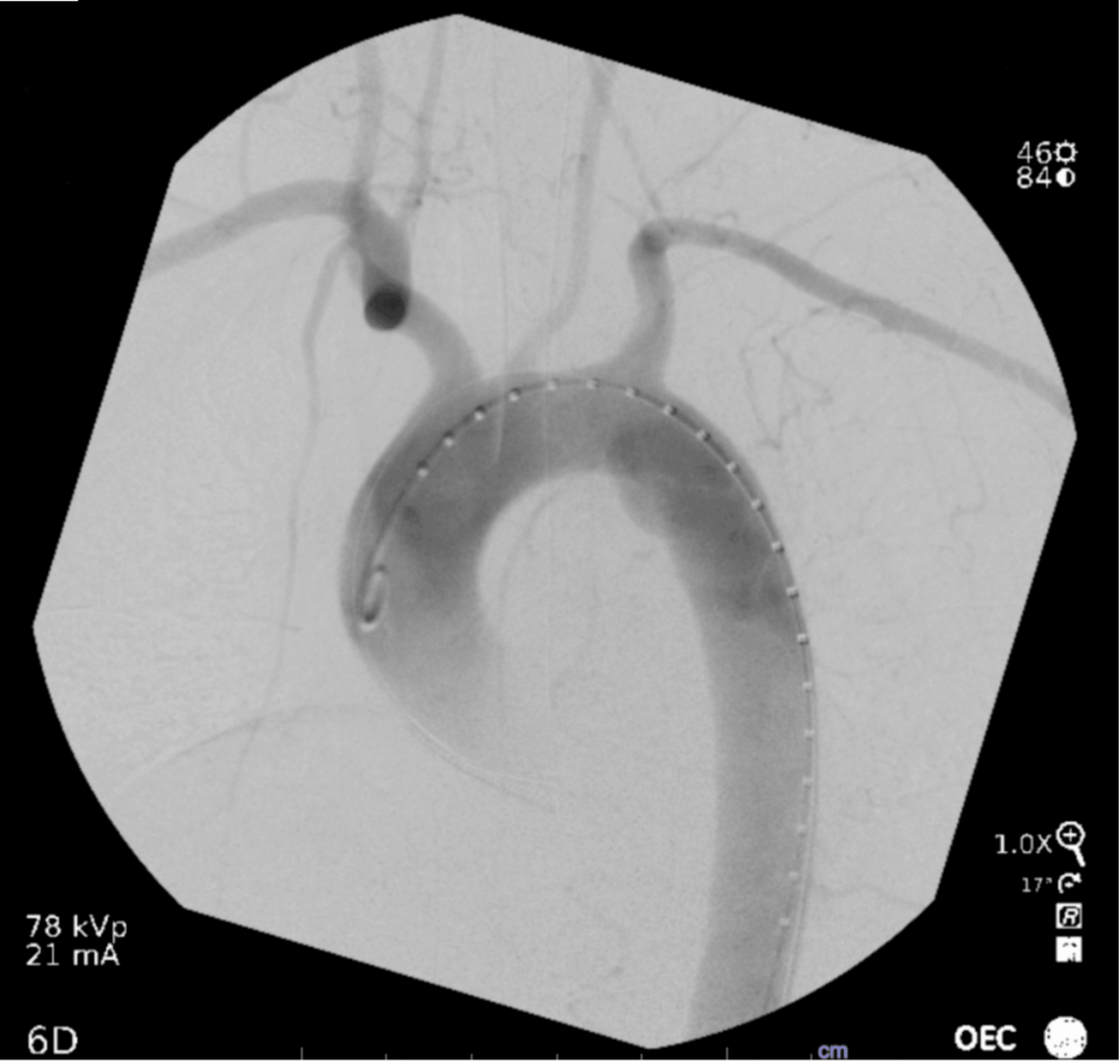
CT angiography with contrast visualizing the aortic arch and descending aorta preplacement of the stent.

Our differential diagnoses included thoracic aortic transection, aortic dissection, aortic rupture, rib fractures with associated hemothorax or pneumothorax, thoracic spine fractures with spinal cord injury, and pulmonary laceration. Diagnostic assessments included physical examination findings, hemodynamic monitoring, imaging studies (e.g., chest X-ray (Figure 1), CT of the chest and abdomen (Figures 2, 3), CT angiogram (Figure 4)), and interventional radiology procedures (e.g., angiography for aortic injury).

**Figure 5:**
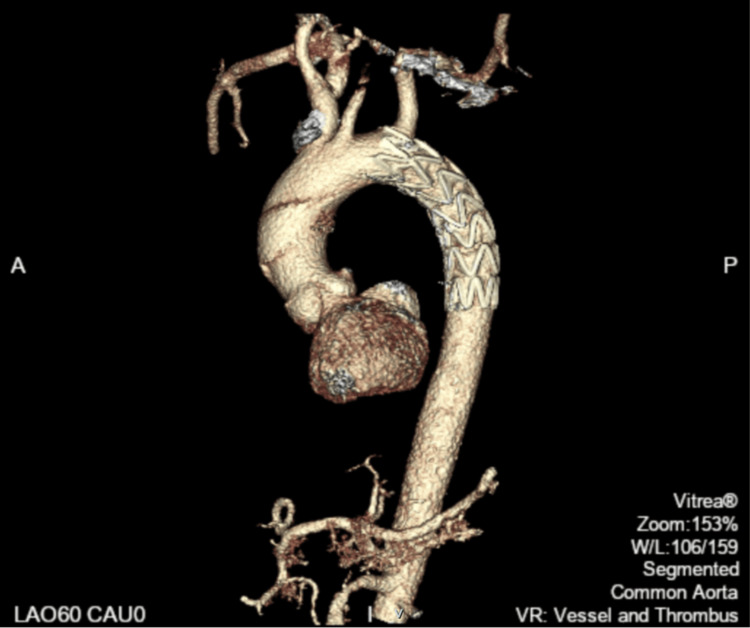
Postoperative 3D reconstruction CT angiography (posterior view) illustrating the aortic repair with a stent. No contrast extravasation can be seen.

Therapeutic interventions included strict blood pressure control with an Esmolol drip, endovascular stenting repair (EVAR) for the aortic injury (Figure 5), open reduction internal fixation for acetabular and rib fractures, intercostal nerve cryoablation, intubation, and mechanical ventilation. Following surgical interventions and stabilization of respiratory status, the patient demonstrated significant improvement with continued progress under proper pain control. The patient was discharged to a rehabilitation center.

## Discussion

Aortic transection is a life-threatening condition characterized by tears in the inner layer of the aorta that lead to the formation of growing hematoma in the intima-media space. It can be caused by penetrating damage, although blunt trauma is the most prevalent cause [2]. There are no clinical findings that are unique to aortic injury [6]. However, common symptoms include hypotension, changed mental state, and outward signs of trauma [7]. A large percentage of individuals who have a whole thoracic aortic transection pass away before reaching the emergency room [8,9]. Following severe aortic damage, a clinician’s strong index of suspicion, quick diagnosis, and efficient treatment are critical to a patient’s life [2,10].

Our case highlights the complex management of a 50-year-old male involved in a high-speed motor vehicle collision resulting in multiple traumatic injuries, including a descending thoracic aortic transection, rib fractures, acetabular fractures, and pelvic fractures. Through a multidisciplinary approach involving trauma surgery, interventional radiology, orthopedic surgery, and critical care, the patient received timely and comprehensive care that ultimately led to a favorable outcome. The mechanism of injury in this case, involving a high-speed collision with a semi-truck, underscores the severity and complexity of the patient’s injuries. The initial presentation with tachycardia and hypotension raised concerns for internal hemorrhage and prompted expedited evaluation and management upon arrival at the emergency department.

Although the diagnosis of traumatic aortic injury is difficult, it can be assisted with diverse imaging tools, such as chest X-ray, FAST, and CT. An efficient and quick method for the early diagnosis of aortic damage is a chest X-ray. Mediastinal enlargement is the most notable symptom [11]. Chest X-rays, however, are unable to offer enough sensitivity and specificity. According to Woodring et al., between 7.3% and 44% of individuals with aortic damage had normal mediastinum upon presentation [12]. As a result, an aortic dissection cannot be entirely ruled out by a regular chest X-ray. Although FAST has a relatively low sensitivity and specificity for diagnosing individuals with aortic dissection, it can also quickly and effectively detect mediastinal widening [13]. The gold standard for diagnosis of traumatic aortic injuries is CT of the chest, especially CT angiography of the thoracic and abdominal aorta, which achieves a sensitivity of nearly 100% [14]. Chest CT must be used as soon as there is a suspicion of a traumatic aortic injury, particularly in individuals who have had abdominal or chest injuries from a car accident [13]. In our case, diagnostic challenges were encountered in identifying the full extent of the patient’s injuries, particularly the aortic transection, which required advanced imaging modalities such as CT angiography for accurate diagnosis. Prompt recognition of aortic injury was crucial, as untreated thoracic aortic transections carry a high mortality rate.

The management approach focused on rapid stabilization and strict blood pressure control to minimize the risk of further aortic injury or rupture. For all age groups, the Society for Vascular Surgery suggests that EVAR should be chosen over open repair if the anatomy is favorable. Endovascular repair has been associated with lower rates of morbidity, mortality, and spinal cord ischemia, as well as more rapid hospital discharge [15]. Endovascular stenting repair (EVAR) was successfully performed to address the aortic transection, demonstrating the effectiveness of minimally invasive techniques in managing complex vascular injuries.

## Conclusions

This case highlights the importance of rapid and accurate diagnosis in trauma settings. The successful application of EVAR in managing traumatic aortic injuries demonstrates its potential as a life-saving intervention. The multidisciplinary approach was crucial in addressing the complexity of the patient’s injuries, illustrating the challenges and advancements in the management of polytrauma patients.
